# Guianensin, a *Simulium guianense* salivary protein, has broad anti-hemostatic and anti-inflammatory properties

**DOI:** 10.3389/fimmu.2023.1163367

**Published:** 2023-07-03

**Authors:** Paola Carolina Valenzuela-Leon, Andrezza Campos Chagas, Ines Martin-Martin, Adeline E. Williams, Markus Berger, Gaurav Shrivastava, Andrew S. Paige, Michalis Kotsyfakis, Lucas Tirloni, Eric Calvo

**Affiliations:** ^1^ Laboratory of Malaria and Vector Research, National Institutes of Health, Bethesda, MD, United States; ^2^ Tick-Pathogen Transmission Unit, Laboratory of Bacteriology, National Institute of Allergy and Infectious Diseases, Hamilton, MT, United States; ^3^ Laboratory of Genomics and Proteomics of Disease Vectors, Institute of Parasitology, Biology Centre, Czech Academy of Sciences, Ceske Budejovice, Czechia; ^4^ Institute of Molecular Biology and Biotechnology, Foundation for Research and Technology-Hellas, Heraklion, Greece

**Keywords:** blood feeding, black fly, saliva, arthropod, enzyme inhibitor, anticoagulant

## Abstract

**Background:**

Salivary glands from blood-feeding arthropods secrete several molecules that inhibit mammalian hemostasis and facilitate blood feeding and pathogen transmission. The salivary functions from *Simulium guianense*, the main vector of Onchocerciasis in South America, remain largely understudied. Here, we have characterized a salivary protease inhibitor (Guianensin) from the blackfly *Simulium guianense*.

**Materials and methods:**

A combination of bioinformatic and biophysical analyses, recombinant protein production, in vitro and in vivo experiments were utilized to characterize the molecula mechanism of action of Guianensin. Kinetics of Guianensin interaction with proteases involved in vertebrate inflammation and coagulation were carried out by surface plasmon resonance and isothermal titration calorimetry. Plasma recalcification and coagulometry and tail bleeding assays were performed to understand the role of Guianensin in coagulation.

**Results:**

Guianensin was identified in the sialotranscriptome of adult *S. guianense* flies and belongs to the Kunitz domain of protease inhibitors. It targets various serine proteases involved in hemostasis and inflammation. Binding to these enzymes is highly specific to the catalytic site and is not detectable for their zymogens, the catalytic site-blocked human coagulation factor Xa (FXa), or thrombin. Accordingly, Guianensin significantly increased both PT (Prothrombin time) and aPTT (Activated partial thromboplastin time) in human plasma and consequently increased blood clotting time *ex vivo*. Guianensin also inhibited prothrombinase activity on endothelial cells. We show that Guianensin acts as a potent anti-inflammatory molecule on FXa-induced paw edema formation in mice.

**Conclusion:**

The information generated by this work highlights the biological functionality of Guianensin as an antithrombotic and anti-inflammatory protein that may play significant roles in blood feeding and pathogen transmission.

## 1 Introduction

Black flies (Diptera: *Simuliidae*) are critical disease vectors that transmit pathogens to humans and livestock during blood feeding (1). In addition to allergic reactions and erythema, the bite of adult female black flies can cause deadly diseases through the transmission of parasites and viruses (2, 3). Human river blindness or Onchocerciasis is a critical disease transmitted by black flies; it is a leading cause of blindness in the developing world, resulting from the infection of filarial parasitic nematode *Onchocerca volvulus* (4, 5). Those most at risk of Onchocerciasis live in rural areas of developing countries, making treatment and preventive efforts difficult (6). There is no vaccine available to prevent Onchocerciasis, and there is evidence of emerging resistance to the only available treatment, the anti-parasitic drug Ivermectin (5). Therefore, vector-based control strategies offer an attractive approach to disease prevention (7). Most research has focused on the only species of black fly that has been successfully colonized in the laboratory, *Simulium vittatum* (8). However, *Simulium guianense* is the primary vector of *O. volvulus* in the Latin American endemic region on the border of Brazil and Venezuela (9).

In order to achieve a successful blood meal, hematophagous arthropods must overcome a variety of host defenses such as hemostasis, fibrinolysis, inflammation, and immune responses initiated by tissue damage at the bite site (10, 11). The saliva of blood-feeding arthropods contains a suite of bioactive compounds that counteract host defenses, which may enhance pathogen transmission; thus, they present potential targets for vaccines and novel therapeutic tools (12–15). During a bite, host tissue damage initiates the three branches of mammalian hemostasis: blood coagulation, vasoconstriction, and platelet aggregation (16). These processes are complex and interconnected, relying heavily on cascades of serine proteases to prevent blood loss (17, 18). Coagulation consists of both intrinsic and extrinsic cascades that share a common pathway through the activation of factor X (FX), ultimately leading to the creation of a fibrin meshwork that stabilizes the platelet plug (19). The intrinsic pathway is further connected to the kallikrein-kinin system, which uses the serine protease kallikrein to produce bradykinin and induces vasodilation and inflammation (20). Interestingly, bradykinin can also trigger pain and itch (21). Fibrinolysis also uses the activation of the serine protease plasmin to regulate the degradation of the fibrin mesh and coagulation (22).

Hematophagous arthropods have evolved Kunitz salivary proteins with broad anti-hemostatic and anti-inflammatory activities (2, 10, 23). Kunitz family proteins are reversible, competitive inhibitors of serine proteases that disrupt host defense systems, including hemostasis and inflammation (24, 25). Kunitz protease inhibitors contain at least one Kunitz domain, which usually consists of a 60 amino acid peptide chain stabilized by three disulfide bridges between cysteine residues (26). Canonical Kunitz inhibition is characterized by a tight interaction between the inhibitor and the protease, similar to the enzyme-substrate Michaelis complex (26). The inhibitor directly binds to the active site of the protease, blocking catalytic activity (27). Structural similarities between common serine proteases allow a single Kunitz inhibitor to have activity against a broad range of enzymes, thus providing a multifunctional role in inhibiting a variety of host processes (25, 28). The anticoagulant activity of *S. guianense* salivary gland homogenate has been characterized, but the specific molecules involved have not yet been identified (9). The sialome of *S. guianense* described proteins in the salivary glands with potential importance for blood feeding and disease transmission, and a secreted Kunitz-type protein was identified that we, henceforth, call Guianensin (29). In this study, we present a detailed description of this Kunitz inhibitor with broad anti-hemostatic and anti-inflammatory properties both *in vitro* and *in vivo*. These findings provide useful insights into a salivary gland protein that inhibits a variety of host defenses, which would facilitate successful blood meal acquisition.

## 2 Materials and methods

### 2.1 Reagents

Purified human factors XIIa, Xa, and thrombin were purchased from Haematologic Technologies Inc. (Essex Junction, VT, USA). Chromogenic substrates S-2222 (Methoxicarbonil-D-cyclohexylglicil-glicil-arginine-para-nitroanilide acetate) and S-2238 (H-D-hexahidrotirosol-alanil-arginine-para-nitroanilide diacetate) were obtained from Diapharma Group Inc. (Westchester, OH, USA). Cathepsin G, human coagulation factor FXIa, urokinase plasminogen activator, and tissue plasminogen activator were obtained from Molecular Innovation (Southfield, MI, USA). Plasmin, thrombin, α-chymotrypsin, chymase, control plasma, lipopolysaccharide (LPS, *E. coli* O111:B4), and rabbit thromboplastin solution were obtained from Sigma-Aldrich (St. Louis, MO, USA). Trypsin was obtained from Roche Molecular Biochemicals (Indianapolis, IN, USA), and elastase was purchased from Elastin Products Company, Inc. (Owensville, MO, USA). Kallikrein was obtained from Fitzgerald Industries International (Concord, MA, USA), Proteinase 3 was purchased from Merck-Millipore, and matriptase from R&D Systems (Minneapolis, MN, USA). Human-purified fibrinogen and prothrombin-deficient plasma were from Diagnostica Stago (Parsippany, NJ, USA). To evaluate the cleavage of the protease-activated receptor 2 (PAR-2) and for endothelial cell prothrombinase experiments, purified factor Va (FVa), prothrombin, factor X (FX), factor Xa (FXa), and endothelial culture media (MCDB 131) were purchased from Thermo Fischer Scientific (Waltham, MA, USA). To measure the protease activity by fluorescence resonance energy transfer (FRET), substrates based on the mouse PAR-2 sequence (NSKGRSLIGR) were acquired from Anaspec (Fremont, CA, USA).

### 2.2 Three-dimensional modeling and amino acid alignment

The amino acid sequence of Guianensin was obtained from the sialotranscriptome of *S. guianense* (29). SignalP 4.1 server was used to detect and remove the signal peptide of the sequence. The Guianensin structure was modeled using the I-TASSER software (30). The sequence coordinates were loaded onto PyMOL to generate the figure. Protein sequences were aligned against other Kunitz inhibitors available on the NCBI database: (*Simulium vittatum* (EU930300.1), *Simulium nigrimanum* (ACZ28223), *Zeugodacus cucurbitae* (XP_011191995), *Ixodes scapularis* (XP_029848488), *Pseudonaja textilis* (Q90WA1), *Vipera ammodytes ammodytes* (AMH40733), and *Homo sapiens* (ABP02055).

### 2.3 Cloning and expression of Guianensin

The mature peptide of Guianensin was chemically synthetized containing two restriction enzyme sites (NdeI 3’end and XhoI 5’-end) and a 6x-His tag before the stop codon (Biobasic Inc., Canada). The synthetic gene coding for mature Guianensin was optimized for bacterial expression and subcloned into pET17b (Invitrogen Life Technologies, San Diego, CA, USA). Chemically competent BL21(DE3) pLysS cells were transformed with the pET17b-Guianensin plasmid and cultured in 250 µL of SOC medium (0.5% Yeast extract, 2% Tryptone, 0.01 M NaCl, 0.0025 M KCl, 0.01 M MgCl_2_, 0.01 M MgSO_4_, and 0.02 M Glucose) according to the manufacturer’s instructions (Invitrogen Life Technologies). The cells were propagated on agar plates supplemented with ampicillin (10 µg/mL) and chloramphenicol (50 µg/mL) at 37°C. Individual colonies were expanded in 1L of *Escherichia coli* culture following the same protocol and antibiotic concentrations described above until they reached the O.D of 0.8 at 37°C and shaking at 2,500 RPM. Bacterial growth was induced with 0.1 mM final concentration of isopropyl-ß-d-thiogalactopyranoside (IPTG) overnight at 20°C and shaking at 100 RPM. Bacterial cells were harvested by centrifugation at 6,000 RPM for 20 min and stored at -80°C until protein extraction and purification.

### 2.4 Recombinant protein purification

Cell pellets were solubilized in 100 mL of 20 mM Tris-HCl and 0.15 M NaCl pH 8.0. Cells incubated on ice were lysed by three sonication cycles (30 sec bursts at 300 W using a Branson 450 Sonifier (VWR)). After sonication, the soluble fraction was separated from the cell debris by centrifugation at 12,000 RPM for 30 min. The supernatant was supplemented with 500mM NaCl and 5 mM Imidazole and loaded onto an affinity column (HP-HiTrap Chelating column, GE Healthcare, Piscataway, NJ, USA), followed by size-exclusion chromatography (Superdex200 10/30 column, GE Healthcare). The identity of purified Guianensin was confirmed by Edman degradation. Purified protein was further processed to remove any LPS contaminant and verified using Endosafe nexgen-PTS (Charles River Laboratories, Wilmington, MA, USA) as described in a previous report (31). The concentration of purified Guianensin was estimated by its absorbance at 280 nm using a DeNovix DS-11 spectrometer (DeNovix, Wilmington, DE, USA), using the molar coefficient ϵ280nm: 9690 M-1.cm-1 and A280nm/cm [1mg/mL]).

### 2.5 Circular dichroism spectroscopy

For circular dichroism spectroscopy **(**CD) analyses, 0.1 mg/mL of purified Guianensin in 20 mM Tris-HCL 150 mM and NaCl pH 8.0 was used. Continuous measurements with a pitch of 0.2 nm were recorded from 200–250 nm wavelengths with a bandwidth of 1 nm. Mean residue ellipticity was calculated with the following equation: (molecular weight of each protein in daltons/((number of amino acids − 1) × θλ))/(10 × pathlength in cm × protein concentration in g/mL). All readings were normalized by subtracting with blank (buffer) mean residue ellipticity. Data were analyzed using CAPITO (32).

### 2.6 Anticoagulant assays: normal plasma recalcification time, prothrombin time, and activated partial thromboplastin time

The recalcification time of human plasma was carried out as described previously (33). Briefly, 30 μl of normal control plasma (Hyphen BioMed) was mixed with equal volumes of serially diluted Guianensin (3 nM – 126 nM). Hepes buffer (10 mM Hepes, 150 mM NaCl, and pH 7.3) was used as a negative control. Coagulation was triggered by adding 30 μl of prewarmed 25 mM CaCl_2_ to each well. Data was collected in a VersaMax plate reader (Molecular Devices, San Jose, CA, USA) at 10 s intervals for over 60 min. Recalcification was determined as the time for each sample to reach 0.025 absorption units at 650 nm. aPTT and PT were measured on a Start 4 Stago coagulometer (Diagnostica Stago, Parsippany, NJ, USA) following the manufacturer’s instructions. Lyophilized citrated normal human control plasma (Hyphen BioMed, Neuville-sur-Oise, France) was reconstituted with distilled water, and 50 µL was transferred to an individual cuvette containing 5 µL of phosphate-buffered saline (PBS) or different concentrations of Guianensin (200 nM – 3.125 nM, prepared in PBS). Each reaction was incubated for 2 min at 37°C. For aPTT, the mixture was supplemented with 50 µL of prewarmed aPTT reagent followed by 3 min of incubation. Coagulation was initiated by the addition of 50 µL of CaCl_2_ (25 mM final concentration). For the PT assay, 100 µL of Neoplastine CI Plus reagent (Diagnostica Stago) was added to the sample to initiate the clot formation. All experiments were carried out in duplicate.

### 2.7 FXa and thrombin chromogenic substrate assay

The role of Guianensin on two common points (Factor Xa activation and thrombin formation) of the coagulation pathway was investigated. In a 96-well plate, 25 µL of TBS (0.025 M Tris, 0.15 mM NaCl, and pH 7.4) or Guianensin in TBS at 83 µg/mL or 57 µg/mL was added to each well, in triplicate, for FXa or thrombin assay, respectively. Seventy-five microliters of FXa (0.5 nM) or thrombin (0.1 nM) solution were added to the wells. FXa was prepared in TBS supplemented with 0.5% BSA and 5 mM CaCl_2_. Calcium chloride was not used for thrombin preparation. Then, samples were incubated for 15 min at 37°C followed by the addition of 5 µL of chromogenic substrates (S-2222 for FXa or S-2238 for thrombin) at final concentrations of 5 mM, with a final reaction volume of 105 µL. Absorbance at 405 nm was measured on a VersaMax microplate reader (Molecular Devices, USA) at 37°C, with reads every 10 sec for 1h.

### 2.8 Prothrombinase assembly assay

Factor Xa inhibition properties of Guianensin were evaluated regarding its role to interfere in the activation of the prothrombin complex by human FXa. The assay was performed as described previously (34). Briefly, all samples were prepared in a TBS-Ca buffer solution (0.02 M Tris, 0.15 mM NaCl, 0.005mM CaCl_2_, 0.3% [w/v] BSA, and pH 7.5). Guianensin (125 pM–0 pM) was incubated with FX (10 nM final concentration) for 20 min at RT. Cascade activation was followed by the addition of human factor Va (1 nM) and phosphatidylcholine and phosphatidylserine (PC/PS) at 10 µM with an incubation time of 5 min. Prothrombinase complex was triggered by the addition of human prothrombin (1.4 nM), and aliquots of 25 µL was collected every other minute and transferred to a new plate containing stop solution (0.02 M Tris, 0.15 mM NaCl, 0.02 M EDTA, 0.1% BSA, and pH 7.5). Twenty-five microliters of S2238 (312.5 µM) were added to each collected aliquot. A kinetic evaluation of the effect of Guianensin in prothrombin assembling was assessed by measurement of absorbance at 405 nm at 37°C during 15 min with 11-sec intervals between reads using a VersaMax microplate reader (Molecular Devices, USA).

### 2.9 Serine protease inhibition screening

Guianensin was screened against a broad panel of various serine proteases involved in blood coagulation and inflammation pathways. The assay was performed as described previously (35) using a panel of 16 serine proteins at specific final concentrations: Plasmin (1.6 nM); Cathepsin G (8.8 nM); Elastase (60 pM); Factor XIa (60 pM); b-tryptase (10 pM); Kallikrein (40 pM); Factor Xa (500 pM); Proteinase 3 (1.3 nM); and t-PA (12 pM). The specification of each enzyme is described in Section 1.1. The IC_50_ (nM) and the *Ki* (nM) of each interaction were calculated as described elsewhere (34).

### 2.10 Surface plasmon resonance

Affinity experiments were carried out by surface plasmon resonance (SPR) using an SPR T200 instrument (GE Healthcare). Materials (CM5 sensor chip, amine coupling immobilization kit, and reagents/buffers) were obtained from GE Healthcare. Guianensin was immobilized at 1250 RU on a CM5 sensor chip by the amine coupling method followed by ethanolamine blocking of empty sites. An additional flow cell on the same sensor chip was immobilized with a running buffer only and served as a non-specific binding parameter during the analysis. The sensor was regenerated with 10 mM glycine-HCl pH 2.0 after each cycle. A blank sensogram, obtained from running buffer only as an analyte, was used to subtract the bulk refractive index background against running curves. Biacore T200 Evaluation Software package was used for the analysis. The analytes investigated included human FXIa; human FXa; human FIXa; Plasmin; Kallikrein; zymogens (Protein Z); Gla-domainless factor Xa (hGla (–)), which is a truncated form of factor Xa, human factor Xa, bovine factor Xa, mouse factor Xa, human ß-factor Xa (hß), and the human factor Xa that had the catalytic site blocked (hDEGR). A kinetic assay was performed for each confirmed molecular partner of Guianensin (Kallikrein, FXIa, FXIIa, FIXa, and Plasmin) with a 500 nM starting concentration and six serial dilutions. Binding running parameters were set at a contact time of 180 sec, a flow rate of 30 µl/min, and 600 sec for dissociation. The individual association (K_a_) and dissociation (K_d_) rate constants and affinity constant (K_D_) were obtained using a global fitting and 1:1 interaction model using the Biacore T200 Evaluation software.

### 2.11 Isothermal titration calorimetry

Isothermal titration calorimetry experiments were performed using a MicroCal VP-ITC machine (Malvern Panalytical, Westborough, MA, USA). Samples were prepared in TBS buffer (20 mM Tris pH 8.0 and 0.15M NaCl) and degassed using a MicroCal ThermoVac (Malvern Panalytical) prior to loading. Titration experiments were performed by successive injections of FXa at 30 μM into the sample cell containing 3 μM Guianensin. Assays were performed at 30°C, and measured heats were converted to enthalpies and analyzed by fitting to a single-site binding model on the Microcal Origin software package version 7 (OriginLab).

### 2.12 Determination of the P1’ site residue in Guianensin

Ten micromolar of Guianensin or FXa alone, or at an equal 1:1 molar ratio, were incubated for 16 h at 37°C in TBS-Ca in a 25 µL total reaction volume. Following the incubation period, 10 µL of LDS buffer (Invitrogen) was added to each sample in the presence of a 4 µL reducing agent (Invitrogen). Samples were loaded onto a 4%–12% SDS-PAGE gel under reducing and non-reducing conditions and blotted to a nitrocellulose membrane. Bands were stained with Coomassie blue without acetic acid. Fragments from the Guianensin cleavage were excised and submitted for N-terminal sequencing for identification.

### 2.13 Tail bleeding assay

All procedures were performed in accordance with the animal study protocol approved by the NIAID Animal Care and Use Committee (ASP#: LMVR3). Sixteen to twenty-week-old female mice (C57BL/6) were anesthetized using a mixture of ketamine and xylazine (90 mg/kg and 10 mg/kg IP) and placed on a warming pad at 37°C. The tail was measured and marked with ink at 1 cm from the tip and transected with a scalpel to lacerate the tail vein. The tail was hung over the edge of the warming pad and immersed in warmed tubes at 37°C containing 100 µl of either water as a control or Guianensin at two different concentrations (0.3 µM and 1 µM), ensuring that the tail tip did not touch the walls or the bottom of the tube. Shed blood was collected for 15 min. The blood volume and concentration of hemoglobin were used to estimate the quantity of shed blood. To measure the hemoglobin content, 900 µl of distilled water was added to each tube to lyse red blood cells and release hemoglobin. Tubes were vortexed, incubated for 10 min at room temperature, and spun down at 12,000 x *g* for 5 min in a bench-top centrifuge. Blood samples were diluted 1:5 in water and added to microtiter flat-bottom 96-well plates (Corning Costar, Corning NY, USA) in technical triplicates. The absorbance was measured at 540 nm in a VersaMax microplate reader (Molecular Devices).

### 2.14 Mouse paw edema

In this study, we utilized 8-week-old female C3H/HeJ mice obtained from Charles River Laboratories and housed in the NIAID Animal Care Facility. Posterior footpads were injected with PBS, Guianensin (10 µg), FXa alone (10 µg), or FXa incubated with Guianensin (10 µg + 10 µg) for 15 min. Intradermal/subcutaneous (s.c.) injections were performed by injecting 50 µl of sample into the footpads using BD Microfine IV needles. Before each injection and at 15, 30, 45, and 60 min, a caliper (Mitutoyo America Corp., Kawasaki, Kanagawa, Japan) was used to measure the thickness of the posterior footpad. In these experiments, statistical significance was determined by two-way ANOVA, using the FXa alone group as the positive reference of inflammation. Statistical differences were set *at p* < 0.05 (*p < 0.05, ***p < 0.001, and ****p < 0.0001).

### 2.15 Coagulation assay on endothelial cells

Human Dermal Microvascular Endothelial Cells (HMEC-1) from ATCC (Catalog number: CRL-3243) were cultured in MCDB 131 medium supplemented with epidermal growth factor (10 ng/mL), hydrocortisone (1 μg/mL), glutamine (10 mM), and fetal bovine serum (10%). The cells were maintained without antibiotics in a 37°C/5% CO_2_ environment following standard cell culture procedures. HMEC-1 cells (1 x 10^3^) were seeded in 96-well plates until they reached a confluent monolayer. The endothelial cells were then activated for 6 and 24 h with 1 μg/mL LPS (O111:B4) in the absence of fetal bovine serum. After the incubation time, culture media was removed, and human plasma (50 μL) was added in the presence or absence of different concentrations of Guianensin (0, 15, 31, 62, 125, 250, and 500 nM) in a final volume of 150 μL. The coagulation process was triggered by 10 mM CaCl_2_, and clot formation kinetics was followed directly on endothelial cell monolayers at 650 nm for 40 min using a Cytation 5 microplate reader (Biotek Instruments, Winooski, VT, USA). Coagulation time (measured in seconds) was defined as the time taken to reach the 0.05 absorbance value (onset Optical Density).

### 2.16 Generation of factor Xa and thrombin on endothelial cell surface

FXa generation was measured on HMEC-1 surfaces through tenase complex activation using human plasma deficient in prothrombin. For this purpose, confluent HMEC-1 cells were incubated with Guianensin (0, 15, 31, 62, 125, 250, and 500 nM) in the presence of prothrombin-deficient plasma (50 µL). Then, the tenase complex was activated by adding thromboplastin (10 µL) in the presence of 10 mM CaCl_2_. The kinetics of FXa formation was measured directly on endothelial cell monolayers at 405 nm in the presence of 2 mM S2222 substrate for 40 min using a Cytation 5 microplate reader (Biotek Instruments, Winooski, VT, USA). In another set of experiments, thrombin generation was also investigated on HMEC-1 by measuring prothrombinase complex activity. In this case, prothrombinase components (20 nM prothrombin, 5 nM FXa, and 1 nM FVa) were assembled on HMEC-1 confluent monolayers and incubated with Guianensin. Thrombin formation was detected after adding human-purified fibrinogen (2 mg/mL, final concentration) in the presence of 10 mM CaCl_2_. Similarly, the kinetics of fibrinocoagulation was followed at 650 nm in the Cytation 5 microplate reader (Biotek Instruments, Winooski, VT, USA).

### 2.17 PAR-2 peptide cleavage analysis

FXa cleavage of PAR-2 peptide in the presence of Guianensin was performed as described previously (36). FXa (10 nM) was incubated with Guianensin (0, 15, 31, 62, 125, 250, and 500 nM) at 37°C in reaction media containing 20 mM Tris-HCl, 150 mM NaCl, and Tween 20 0.01% (pH 7.4). After 15 min, a FRET-substrate based on the mouse PAR-2 sequence (NSKGRSLIGR) conjugated with 7-methoxycoumarin-4-acetic acid and the quenching group Dnp (2,4-DNP) was added to a final concentration of 2 mM. The peptide hydrolysis rate was followed at 320 nm excitation and 420 nm emission in kinetic mode at 30°C using a Synergy H1 microplate reader (Biotek Instruments, Winooski, VT, USA). PAR-2 cleavage products generated by FXa in the presence or absence of Guianensin were also analyzed by electrospray ionization-mass spectrometry (ESI-MS). A Q Exactive Plus mass spectrometer was used at a resolution of 280,000, a spray voltage of 3.5 kV, and a capillary temperature of 32 °C. Samples were desalted by C18 Zip-tip (Millipore, Burlington, MA, USA) and dissolved in 100 μL of reconstitution buffer (50% ACN, 49% H_2_O, and 1% FA). Samples were introduced using a syringe pump (Thermo Fisher Scientific, Waltham, MA, USA) with a flow rate of 5 μL/min. Xtract algorithm of the Freestyle (Thermo Fischer Scientific, version 1.5) was used to deconvolute the raw data. Deconvolution parameters were set as output mass, MH+; charge range, 1–5; minimum number detected charge, 2.

### 2.18 Data analysis

The data are presented as means ± SE, and significant differences were analyzed by one-way ANOVA followed by an unpaired *t*-test with Bonferroni correction for multiple comparisons. *P*-values of 0.05 were considered significant. Statistical analyses were performed using GraphPad Prism (GraphPad Software Inc., San Diego, CA, USA) software.

## 3 Results

### 3.1 Initial characterization of Guianensin

**Figure 1:**
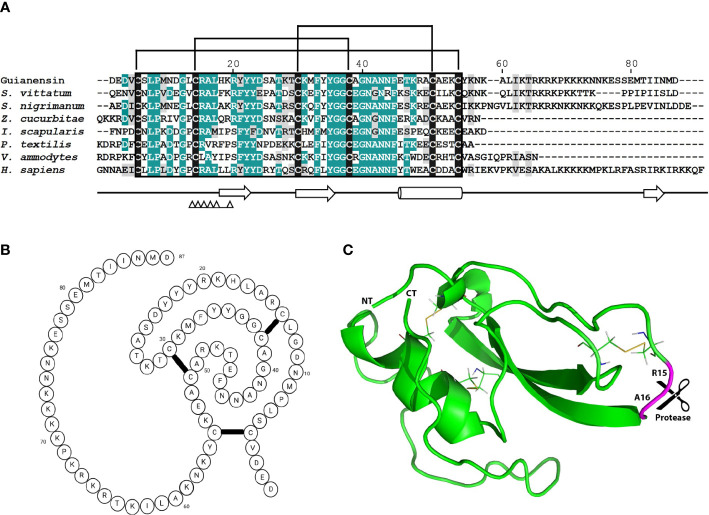
The comparison of mature Guianensin amino acid sequence with seven other single-domain Kunitz protease inhibitors. **(A)** Sequence alignment of single-domain Kunitz protease inhibitors from *Simulium vittatum* (EU930300.1), *Simulium nigrimanum* (ACZ28223), *Zeugodacus cucurbitae* (XP_011191995), *Ixodes scapularis* (XP_029848488), *Pseudonaja textilis* (Q90WA1), *Vipera ammodytes ammodytes* (AMH40733), and *Homo sapiens* (ABP02055). Conserved amino acids are shaded in teal, and similar residues are shaded in gray. Conserved cysteine residues are highlighted in black, and brackets denote disulfide bonds. Amino acid (AA) numbers correspond to the mature Guianensin sequence. The conserved Kunitz domain is shaded in black, and AAs within the trypsin interaction site are marked by triangles. Sequences were aligned using CLUSTAL and refined using the Boxshade server. **(B)** The predicted folding structure figure (created with BioRender.com) for Guianensin shows three disulfide bonds formed between the six cysteine residues present in the protein. **(C)** The tertiary structure model of Guianensin was constructed using the coordinates generated by I-TASSER and visualized in PyMol software. α-helices are indicated by cylinders and β-strands by arrows. The expected P1P1′ site (R15 and A16) is marked.

The previously described sialome of female adult *S. guianense* revealed the presence of a typical single Kunitz protein deducted from three expressed sequence tags (ESTs). The cDNA of *Guianensin* (GeneBank: JI626169) has an open reading frame of 321 base pairs (bp) that encodes for a protein of 106 amino acids (AA). It has a predicted signal peptide (37) of 19 AA, indicative of secretion. The calculated mature Guianensin protein (GenBank: AEB96404) has a molecular weight (MW) of 10074.65 Da and an isoelectric point of 9.45. Two tryptic peptides obtained by MS/MS matching Guianensin were identified by SDS-PAGE, followed by Liquid Chromatography with tandem mass spectrometry (LC/MS/MS). The protein band corresponding to native Guianensin agrees with the calculated MW, suggesting that Guianensin has no significant post-translational modifications *in vivo* (29). Alignment of Guianensin with other proteins of the same family shows the presence of the conserved trypsin interaction (FxxGGCxxxxxxFxxxxxC) site found in most single Kunitz domain proteins characterized to date (**Figure 1A**). A three-dimensional model of Guianensin was generated using the I-TASSER software. The overall 3D modeled structure of Guianensin shows six cysteine residues stabilizing the structure by forming three putative disulfide bonds (**Figures 1B, C**). Guianensin appears to display an extended and accessible reactive-site loop, with complementarity to the protease catalytic site, which might be responsible for its inhibitory activity. The overall reactive-center loop geometry with other Kunitz inhibitors suggests that Guianensin might have a similar mechanism of action on serine proteases described elsewhere (38). Recombinant Guianensin was purified from the soluble fraction and runs as a single peak at the expected mass by size-exclusion chromatography, suggesting that this salivary inhibitor is monomeric (**Supplementary Figure S1**). CD analysis of Guianensin shows that it comprises mainly irregular structures (55%) followed by α-helices (12%) and 32% of β-sheets (**Supplementary Figure S1**). The predicted P1 site residue (Arg15) was experimentally confirmed for factor Xa (FXa) (**Supplementary Figure S2**).

### 3.2 Guianensin is a FXa-directed anticoagulant

**Figure 2:**
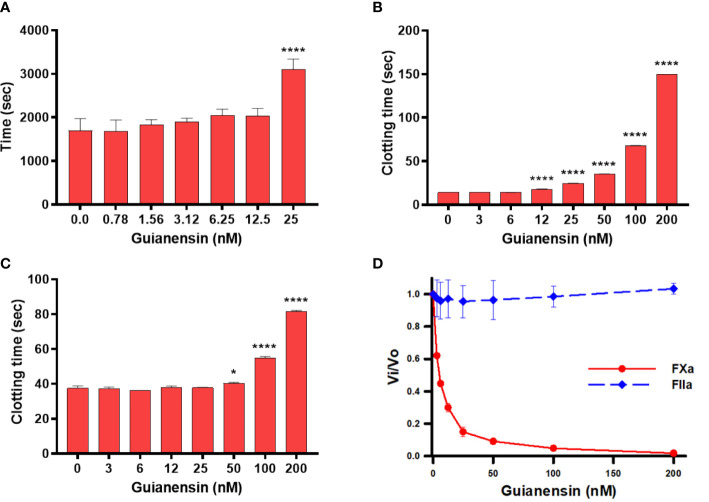
Guianensin has an anticoagulant activity that targets FXa but does not target thrombin. **(A)** Recalcification time was determined using plasma mixed at the indicated concentrations of Guianensin, and reactions were initiated by the addition of CaCl_2._ The involvement of Guianensin in the **(B)** extrinsic and **(C)** intrinsic clotting pathways was tested using PT and aPTT. To analyze the results, we used one-way ANOVA with the control group as a reference. Statistically significant differences are denoted by *p < 0.05, or ****p < 0.0001. **(D)** Amidolytic activity of FXa and FIIa was determined in the presence of increasing concentrations of Guianensin.

Initial experiments using recalcification time of normal plasma confirmed that Guianensin acts as an anticoagulant *in vitro* (**Figure 2A**). We also showed that Guianensin significantly prolonged PT and aPTT (**Figures 2B, C**). These results suggested that Guianensin may inhibit a factor in the common coagulation cascade pathway. Consequently, Guianensin inhibited the amidolytic activity of FXa; however, no thrombin inhibition was observed (**Figure 2D**). These findings are consistent with the anticoagulant activity reported in salivary gland extracts of *S. guianense* (9).

### 3.3 Guianensin is a tight inhibitor of FXa

**Figure 3:**
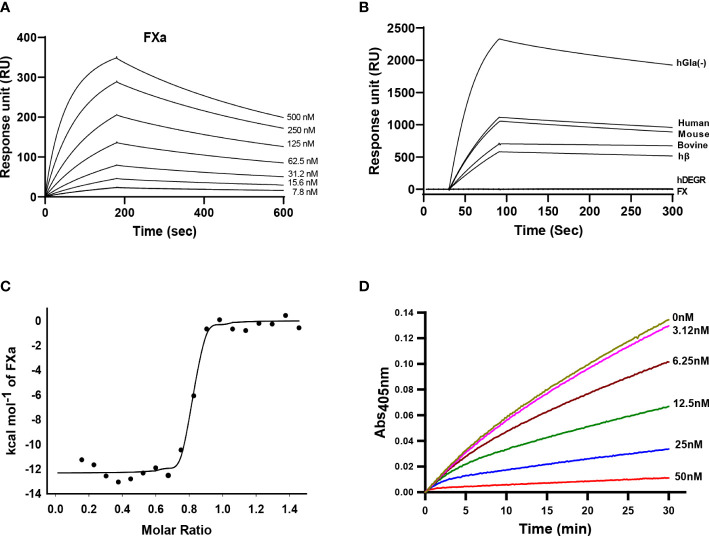
Kinetics of Guianensin interaction with FXa. **(A)** Factor Xa at different concentrations was injected over immobilized Guianensin for 180 seconds. The dissociation of the Guianensin-FXa complex was monitored, and a global 1:1 binding model was used to calculate kinetic parameters. **(B)** Binding analysis of Factor X, hβ, and Factor Xa from human, mouse, and bovine over immobilized Guianensin. We also included modified forms of human FXa (hGla(-) and hDEGR). All analytes were used at a concentration of 100 nM. **(C)** Binding of FXa with Guianensin as measured by isothermal titration calorimetry. The plots represent enthalpies calculated from measured heats (points) versus the molar ratio of FXa to Guianensin in the reaction cell. The data were fitted to a single-site ligand binding model (solid line). The parameters obtained from the fit are shown in **Table 2**. **(D)** The activation of prothrombin complex in the presence of Guianensin. Guianensin at different concentrations was incubated with FXa, FVa, PC/PS, and prothrombin. Aliquots were removed at the plotted times, and the concentration of activated thrombin was determined using the chromogenic substrate S2238.

To investigate the binding interaction between Guianensin and FXa, surface plasmon resonance (SPR) experiments were performed. Typical kinetic sensograms revealed that Guianensin exhibits high affinity binding to FXa (**Figure 3A**; **Table 1**). We also found that Guianensin binds FXa from other species (mouse and bovine), human Gla-domainless, (hGLA (-)), and hβ. No binding was detected with active site-blocked factor Xa (hDEGR), indicating that Guianensin displays reversible, substrate-like binding to the catalytic site of FXa (**Figure 3B**) as proposed for Kunitz-type inhibitors. Through isothermal calorimetry (ITC) assays, we analyzed the stoichiometry and enthalpy of the interaction between FXa and Guianensin. The complex inhibitor-protease was exothermic, with a favorable enthalpy (ΔH) of −12.32 kcal/mol and slightly unfavorable entropy (ΔS = −1.14 cal/mol/deg). Stoichiometry of the binding (*N* = 0.786 ± 0.006 sites) indicates that one molecule of Guianensin binds to one molecule of FXa (**Figure 3C**). The calculated Gibbs free energy (ΔG) of -11.97 kcal indicates that the complex formation occurs spontaneously at a physiological temperature. Thermodynamic parameters are shown in **Table 2**. FXa formation was observed at different starting concentrations of Guianensin over a time course. The kinetic pattern obtained is typical for a slow-binding inhibitor as seen with many peptide inhibitors of serine proteinases (**Figure 3D**). Accordingly, Guianensin also blocked prothrombinase activity *in vitro* (**Supplementary Figure S3**). Taken together, our results indicate that Guianensin is a salivary FXa-directed anticoagulant in *S. guianense* and may account for the anti-FXa activity found in salivary gland extracts previously described for this species (9).

**Table 1:** Surface plasmon resonance analysis of Guianensin-FXa interaction.

K_a_ (m^-1^ s^-1^)	K_d_ (s^-1^)	K_D_ (M)
45050 ± 3606	0.001211 ± 3.111e-005	2.700e-008 ± 2.828e-009

**Table 2:** Thermodynamic parameters of Guianensin-FXa complex measured by isothermal titration calorimetry.

N (sites)	K_D_ (nM)	ΔH (kcal/mol)	ΔS (cal/mol/deg)	ΔG (kcal)
0.786 ± 0.006	2.32 ± 0.42	-12.32 ± 0.022	-1.14	-11.97

### 3.4 Guianensin inhibits other proteases involved in coagulation and inflammation

**Figure 4:**
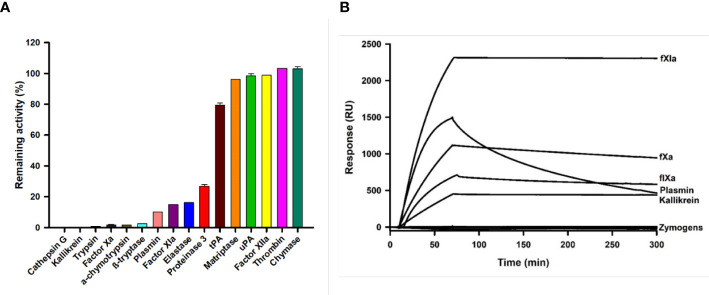
The inhibition of serine proteases activity in the presence of Guianensin. **(A)** Enzyme activities, defined as a percentage of the total activity in the presence of Guianensin at 200 nM, are shown. The graph shows mean ± SEM. u-PA: urokinase-type plasminogen activator; t-PA: tissue plasminogen activator. Each inhibition assay was conducted in triplicate. **(B)** FXIa, FXa, FIXa, Kallikrein, and Plasmin were allowed to flow over immobilized Guianensin using SPR. All analytes were used at 100 nM.

Kunitz family proteins target many serine proteinases involved in coagulation, digestion, and inflammation, among other physiological processes. We also found that Guianensin inhibited other serine proteases that might be relevant for *S. guianense* blood-feeding behavior. Guianensin significantly reduced the activity of cathepsin G, kallikrein, trypsin, α-chymotrypsin, β-tryptase, plasmin, elastase, proteinase 3, and the coagulation factor XIa (**Figure 4A**). The half-maximal inhibitory concentration (IC_50_) and inhibition constant (*Ki*) values for each serine protease were calculated as described previously (39). The inhibition constant (*Ki*) values calculated for each Guianensin-serine protease were in the low nM range, suggesting a strong inhibitory activity against these enzymes (**Supplementary Table 1**). These results were confirmed by SPR (**Figure 4B**). The affinity of Guianensin for FXIa, FIXa, Kallikrein, and Plasmin calculated by SPR was also in the subnanomolar range (**Supplementary Figure 4**). Binding constant values are in **Supplementary Table 2**.

### 3.5 Guianensin inhibits FXa generation and prothrombinase activity in endothelial cells

**Figure 5:**
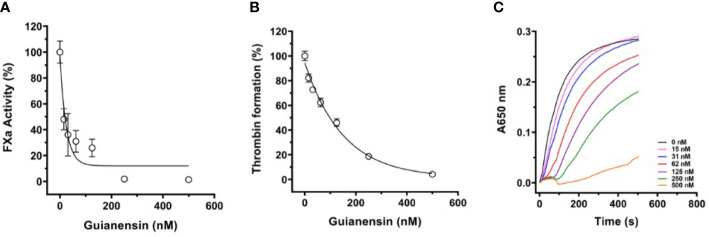
The inhibition of FXa generation and prothrombinase complex activity by Guianensin *in vitro*: **(A)** FXa generation in prothrombin-deficient plasma was measured on endothelial cell monolayers. Human prothrombin-deficient plasma was incubated with confluent HMEC-1 cells in the presence or absence of Guianensin. The tenase complex was then activated by the addition of calcium thromboplastin, and FXa formation was measured in the presence of S2222. Data are presented as mean ± SE of percent FXa activity measured in the absence of Guianensin. **(B)** The prothrombinase complex (20 nM prothrombin, 5 nM FXa, and 1nM FVa) was assembled on HMEC-1 confluent monolayers. After incubation with Guianensin, thrombin formation was measured by the fibrinogen addition. **(C)** The dose-response inhibition curve for thrombin generation and the right panel shows representative curves of kinetic progress for thrombin-induced fibrinocoagulation. Data are presented as mean ± SE of percent thrombin formation measured in the absence of Guianensin.

To study the effects of Guianensin in the context of cell-mediated initiation of blood coagulation, HMEC-1 was stimulated with human prothrombin-deficient plasma in the presence or absence of Guianensin. After 30 min, the tenase complex was activated by the addition of calcium thromboplastin, and FXa formation was measured in the presence of S2222. We observed that increasing concentrations of Guianensin decreased the activity of FXa (**Figure 5A**). We further examined the inhibitory effect of Guianensin on prothrombinase activity in endothelial cells. As shown in **Figures 5B, C**, Guianensin decreased prothrombinase activity in a dose-dependent manner *in vitro*.

### 3.6 Guianensin inhibits FXa cleavage of PAR-2 peptide

**Figure 6:**
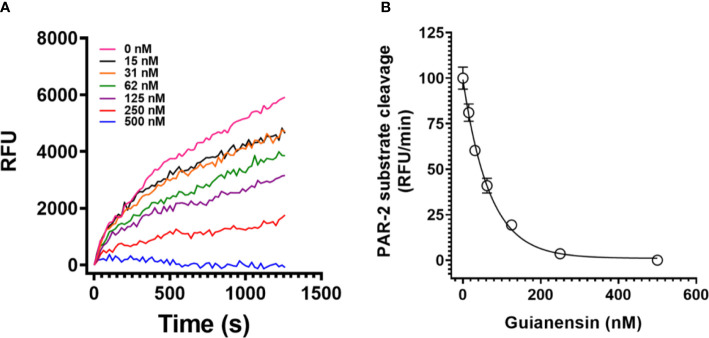
Guianensin dose-dependently inhibits FXa-induced PAR-2 substrate cleavage. **(A)** FXa-induced PAR-2 cleavage was investigated by fluorescence resonance energy transfer (FRET) technology. A FRET-substrate was synthetized based on the mouse PAR-2 (NSKGRSLIGR) sequence containing the fluorescent group MCA (7-methoxycoumarin-4-acetic acid) and the quenching group DNP (2,4-DNP) at the N- and C-terminal ends, respectively. FXa was then incubated with different concentrations of purified Guianensin, and the reaction was triggered by the FRET-substrate addition. The peptide hydrolysis rate was followed at 320 nm excitation and 420 nm emission. **(B)** Representative data for kinetic progress curves and the right panel shows the dose-response inhibition curves of FXa-induced PAR-2 hydrolysis. Data are presented as mean ± SE of the percent of PAR-2 hydrolysis measured in the absence of Guianensin.

PARs can be activated through the action of serine proteases, including FXa. Since Guianensin is a specific inhibitor of FXa, we hypothesized that inhibition of FXa by Guianensin renders this protease unable to activate PAR. Our results show that, in the presence of Guianensin, FXa is unable to cleave PAR-2 *in vitro* (**Figures 6A, B**). We also confirmed these results by a mass spectrometry analysis of PAR-2 peptide treated with FXa in the presence or absence of Guianensin (**Supplementary Figure 5**).

### 3.7 Guianensin prolongs bleeding *ex vivo*

**Figure 7:**
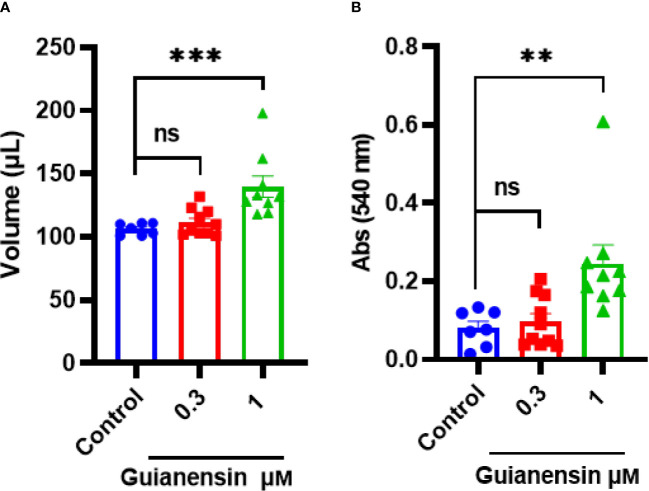
Guianensin impairs host hemostasis *ex vivo*. **(A)** The volume of shed blood after laceration of the tail vein. **(B)** Hemoglobin content is expressed as the mean of the absorbance of technical triplicates at 540 nm ± SEM. Results from at least seven animals are represented for each group. Multiple comparisons were done by one-way ANOVA (***p < 0.001; **p < 0.01; ns: not significant).

To determine any anti-hemostatic effects of Guianensin *ex vivo*, tail bleeding time was measured in mice. Guianensin significantly prolonged coagulation when compared with controls. Guianensin acted as an anticoagulant at the injury site and prevented coagulation, leading to major bleeding, defined as an increase in blood volume and hemoglobin content (**Figures 7A, B**). In addition, Guianensin showed anticoagulant activity on endothelial cells activated with LPS (**Supplementary Figure 6**).

### 3.8 Guianensin inhibits Factor Xa-mediated acute inflammation *in vivo*

**Figure 8:**
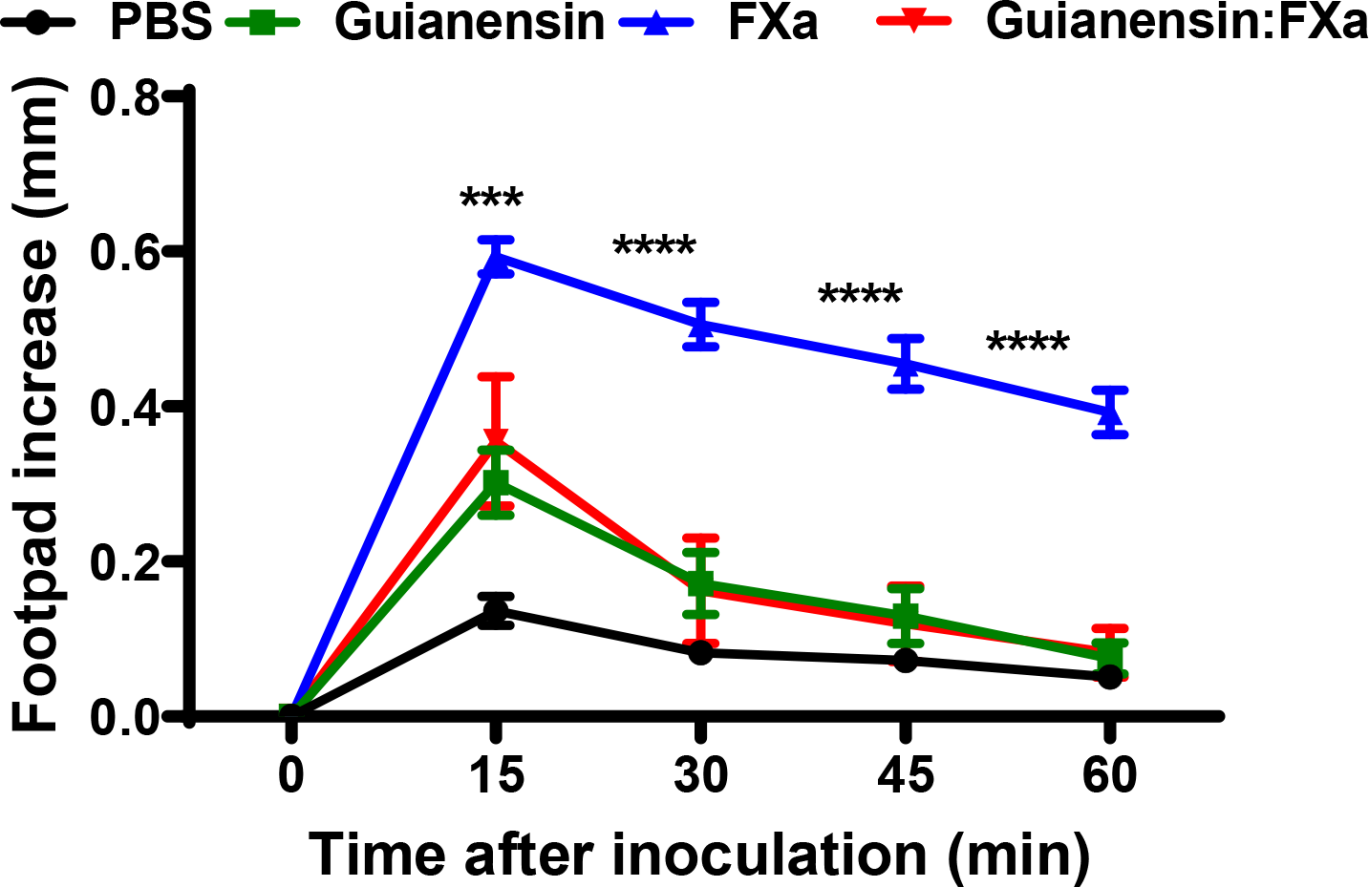
Guianensin blocks the inflammatory effects of FXa. Paw edema was induced by intradermal injection of FXa (10 µg) in the presence of PBS as a negative control or FXa previously incubated with Guianensin at the same concentration. Edema formation was determined using calipers before and after injection of FXa at selected time intervals. Symbols are means ± SEM of five mice per group: ***p < 0.001, or ****p < 0.0001..

Previous studies have shown that subplantar injection of FXa induces paw edema in mice and rats in a time- and dose-dependent manner (36, 40). To assess whether Guianensin also inhibits the inflammatory activity of FXa *in vivo*, 10 µg of FXa was injected either alone in the mouse footpad or in the presence of Guianensin at a molar ratio of 1:1. FXa produced a time-dependent increase in paw edema, which peaked at 15 minutes, and styed significantly inflamed after 1 hour. In the presence of Guianensin, FXa-induced inflammation was significantly reduced, demonstrating its anti-inflammatory activity *in vivo* (**Figure 8**).

## 4 Discussion

Like many blood-feeding arthropods, *S. guianense* flies are anautogenous and require a blood meal for egg development (41). Among the several challenges faced by bloodsucking arthropods, the vertebrate hemostatic response against blood loss represents an important barrier to efficient blood feeding. This feeding behavior can also result in pathogen transmission to their vertebrate hosts. Consequently, these blood feeders have evolved bioactive salivary components that counteract vertebrate hemostasis and inflammation (42). Salivary secretions from black flies contain platelet aggregation inhibitors, vasodilators, anticoagulants, and immunomodulators (2, 3, 10, 14, 25, 43). Salivary gland extracts from female *S. guianense* display anticoagulant activity (9); however, the factor(s) responsible for this activity remained elusive thus far. Bioinformatic surveys of the *S. guianense* salivary transcriptome revealed the presence of a Kunitz-type serine protease inhibitor (29). Kunitz inhibitors are a class of serine protease inhibitors spanning from microbes to mammals. They are competitive inhibitors, acting in a substrate-like manner with a 1:1 stoichiometry with their target protease. These proteins can have single or multiple Kunitz inhibitory domains linked together or are associated with other domain types, and they have been implicated in various physiological processes, including inhibition of blood clotting and inflammation (26). Kunitz inhibitors found in blood-feeding arthropods, as well as in poisonous animals, act as anti-coagulant, anti-microbial, and anti-inflammatory mediators (26).

In this work, we identified a Kunitz-type serine protease inhibitor from *S. guianense* that we named Guianensin. We showed that Guianensin is a salivary anticoagulant that targets coagulation FXa. The lack of binding of Guianensin to FX and the catalytic-blocked FXa indicated that this inhibitor binds directly to the catalytic site of the protease. Guianensin was also active against other serine proteases involved in blood clotting and inflammatory immune responses. Several serine proteases inhibited by Guianensin – neutrophil elastase, cathepsin G, and proteinase 3 – are important for the activation of innate immune responses and for scavenging infectious agents (44). Other serine proteases targeted by Guianensin included α-chymotrypsin, β-tryptase, and trypsin. These proteases can trigger inflammatory responses in the vertebrate host, inducing the expression of chemokines, cytokines, cell surface receptors, and adhesion molecules (27, 45, 46). It is over the damaged endothelium some hemostatic processes and inflammation are activated, and endothelial cells can trigger antithrombotic or prothrombotic events depending on tissue needs (47, 48). Some mechanisms activated on the endothelial cell surface during an injury are the intrinsic tenase and prothrombinase complexes, which enhance the activities of FXa and FIIa, generating enough thrombin to produce large amounts of insoluble fibrin to form the blood clot (49, 50). Guianensin inhibited the generation of FIIa (thrombin) by the tenase complex dose-dependently, both in solution and on endothelial cells. Accordingly, Guianensin increased bleeding time in mice, clearly indicating that this inhibitor may work *in vivo*. In addition to its role in blood coagulation, FXa is also a direct pro-inflammatory molecule acting on PARs (51). PARs, a family of G protein-coupled receptors with seven transmembrane domains, are activated following proteolytic cleavage of their extracellular N-terminus. In fact, PAR activation in a variety of cell types in and around blood vessels is associated with the production of pro-inflammatory cytokines, expression of adhesion molecules, and regulation of vascular tone in smooth muscle cells that mediate contraction migration and proliferation (52–56). We also found that Guianensin was able to inhibit PAR activation in endothelial cells. Furthermore, our experiments in mice showed how Guianensin decreased inflammation induced through the subplantar inoculation of FXa. Combined, we conclude that this salivary inhibitor regulates these pro-inflammatory and procoagulant processes that can be detrimental to the acquisition of a blood meal by *S. guianense.* Salivary secretions suppress the hemostatic system of vertebrate hosts, helping the invertebrate to obtain a blood meal (57–59). Consequently, the presence of Guianensin in the saliva of this species represents an evolutionary adaptation for blood feeding. By inhibiting proteases involved in coagulation and inflammation, Guianensin may reduce the time necessary to successfully feed on vertebrate blood. Awareness from the vertebrate hosts may result in aborted feeding or death of the arthropod. Hence, faster probing and feeding times would reduce the duration of vector-host contact and increase the survival of the feeder.

Our results collectively indicate that Guianensin facilitates blood feeding by disrupting coagulation and interferes with host inflammatory responses. The broad anti-protease activity displayed by Guianensin is not unexpected because Kunitz inhibitors from other blood-sucking arthropods, snakes, and other organisms show similar inhibitory properties. For example, Simukunin, a Kunitz inhibitor from *S. vittatum*, inhibits the activity of multiple proteases, including coagulation factors (25). However, Guianensin shows a stronger inhibition of Kallikrein, Cathepsin G, β-tryptase, and α-chymotrypsin, but a lower activity against elastase. These differences suggest that Simukunin and Guianensin may have different mechanisms of protease inhibition. More structure-function studies will be necessary to elucidate these inhibitor-protease interactions.

We hypothesize that the secretion of Guianensin at the bite site is an evolutionary strategy to block host proteases that could facilitate blood meal acquisition by inhibiting coagulation and inflammation. Future functional studies will be needed to fully understand the role of Guianensin in blood feeding and pathogen transmission by black flies. This work is another example of the complexity of the biology of blood-feeding arthropods and highlights the potential use of vector salivary proteins as therapeutic applications and potential targets for transmission-blocking vaccines.

## Data availability statement

The original contributions presented in the study are included in the article/**Supplementary Material**, further inquiries can be directed to the corresponding author/s.

## Ethics statement

The animal study was reviewed and approved by NIAID Animal Care and Use Committee (ASP#: LMVR3).

## Author contributions

Planned the experiments: PV-L, AC, LT, and EC; performed the experiments: PV-L, AC, IM-M, MK, GS, AW, LT, and EC; analyzed data: PV-L, AC, IM-M, AP, MK, LT, and EC; wrote the paper: PV-L, AP, and EC. All authors contributed to the article and approved the submitted version.

## Author Disclaimer

Because the authors are government employees and this is government work, the work is in the public domain in the United States. Notwithstanding any other agreements, the NIH reserves the right to provide the work to PubMed Central for display and use by the public, and PubMed Central may tag or modify the work consistent with its customary practices. You can establish rights outside of the U.S. subject to a government-use license.
