# Supplementary material for: Guianensin, a Simulium guianense salivary protein, has broad anti-hemostatic and anti-inflammatory properties
Source: Front Immunol. 2023 Jul 3;14:1163367. doi: 10.3389/fimmu.2023.1163367 (PMC10353047; doi:10.3389/fimmu.2023.1163367)
Supplement: Supplementary file 1 [file DataSheet_1.docx]

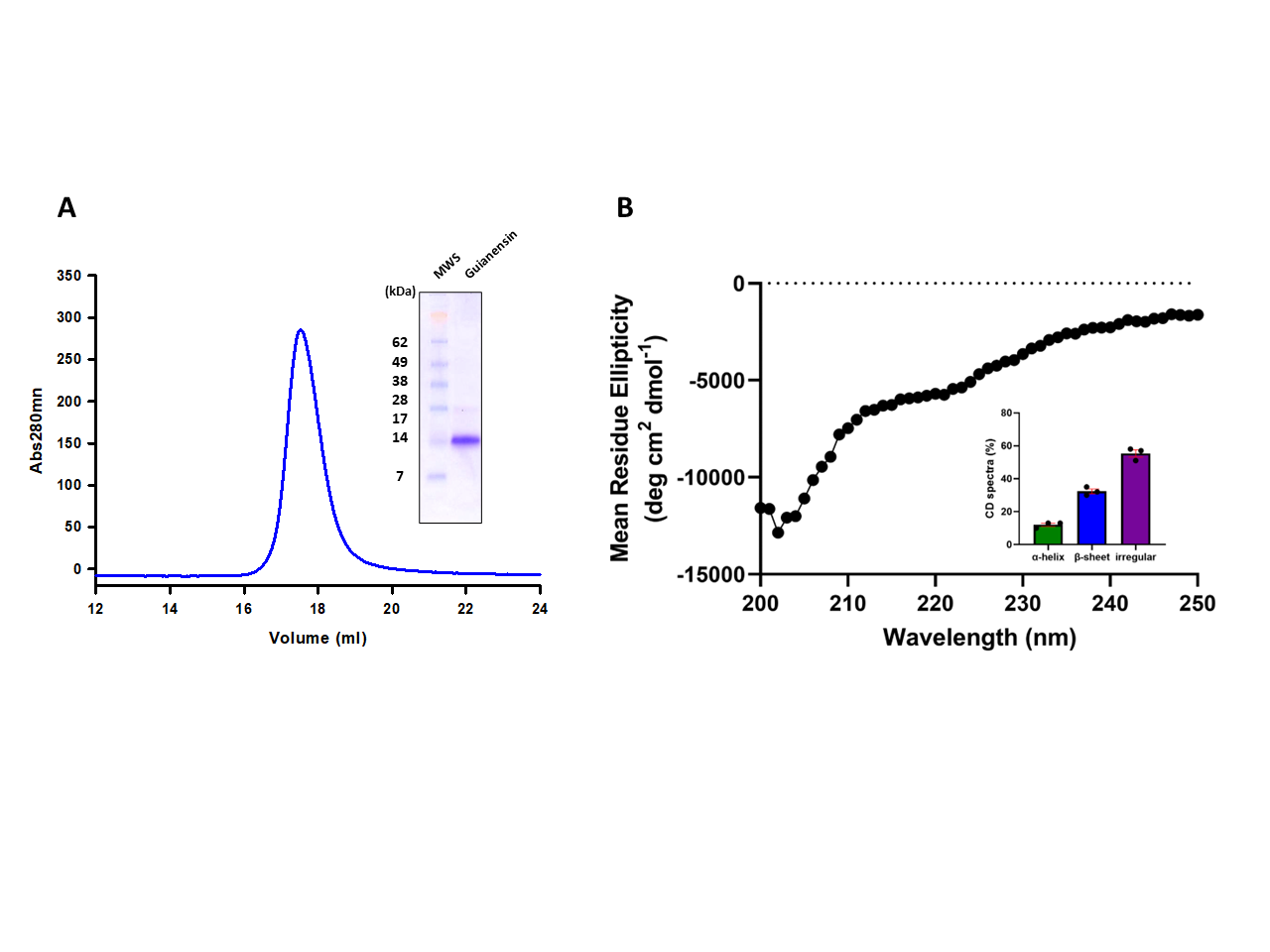


**Supplementary Figure 1: Purification of recombinant Guianensin and circular dichroism analysis.** (A) Recombinant Guianensin was expressed in *E. coli* and purified by affinity and size exclusion chromatographies. Inset shows Coomassie blue stained Guianensin. (B) Circular dichroism (CD) spectra analysis of Guianensin**.** CD spectra curve, by mean residue ellipticity, recorded over 200–250 nm. Inset shows the calculated percentages of secondary structures determined by CD analysis using CAPITO. Red bars indicate mean and standard deviation of three similarity hits based on lowest area differences under the curve.

**
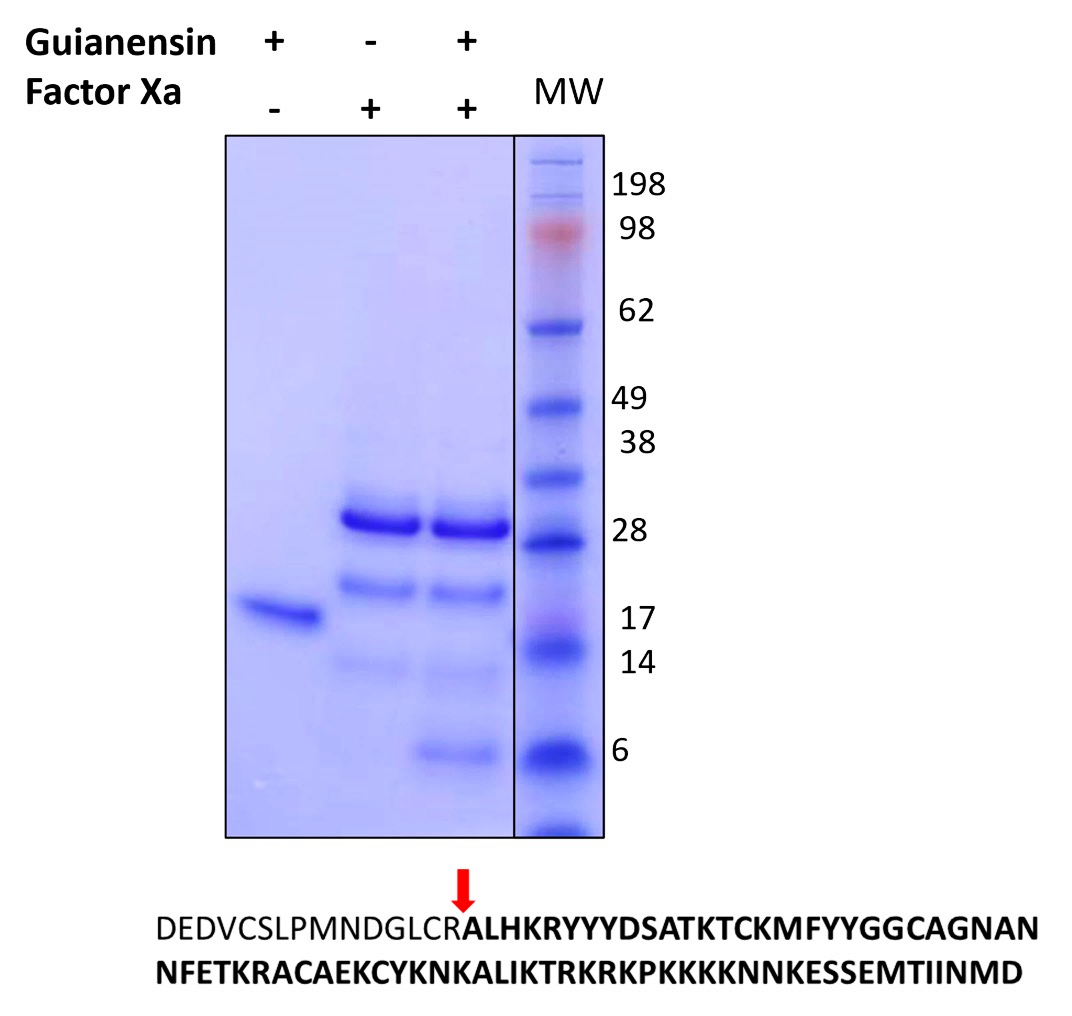
**

**Supplementary Figure 2: Identification of cleavage site of Guianensin by FXa**. To identify the P1 and P1’ residues of Guianensin, 10µg FXa was incubated with Guianensin (1:1 ratio). After 16 hours, the samples were electrophoresed in a 4-12% NuPAGE and stained with Coomassie blue. The cleaved band was submitted to N-terminal sequencing, which revealed Arginine 15 as the P1 residue.


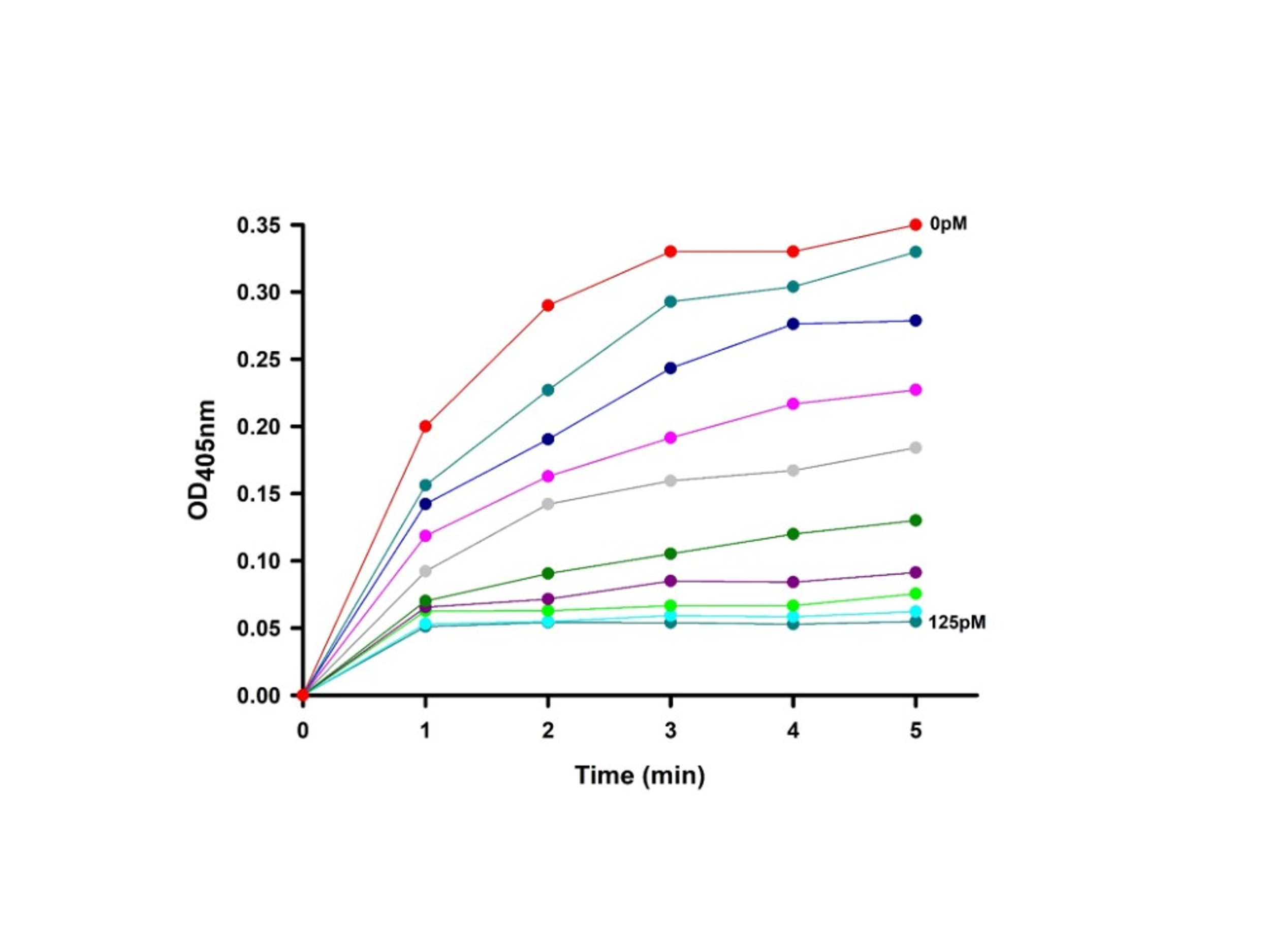


**Supplementary Figure 3: Guianensin inhibits prothrombinase activity.**FXa was incubated with Guianensin at different concentrations (2-fold dilutions), followed by addition of FVa, phosphatidylcholine/phosphatidylserine, and prothrombin in the presence of Ca^2+^ using discontinuous assay. After addition of S-2238, absorbance at 405 nm was recorded at 37°C for 5 min at 11 s intervals using a Thermomax Microplate Reader (Molecular Devices).

**Supplementary table 1: Effect of Guianensin on the activities of various serine proteases.** The enzymatic repertoire tested for inhibition by Guianensin and the concentration of Guianensin at which 50% inhibition of the activity of the targeted serine proteases was achieved (IC_50_) ± S.E. are presented.

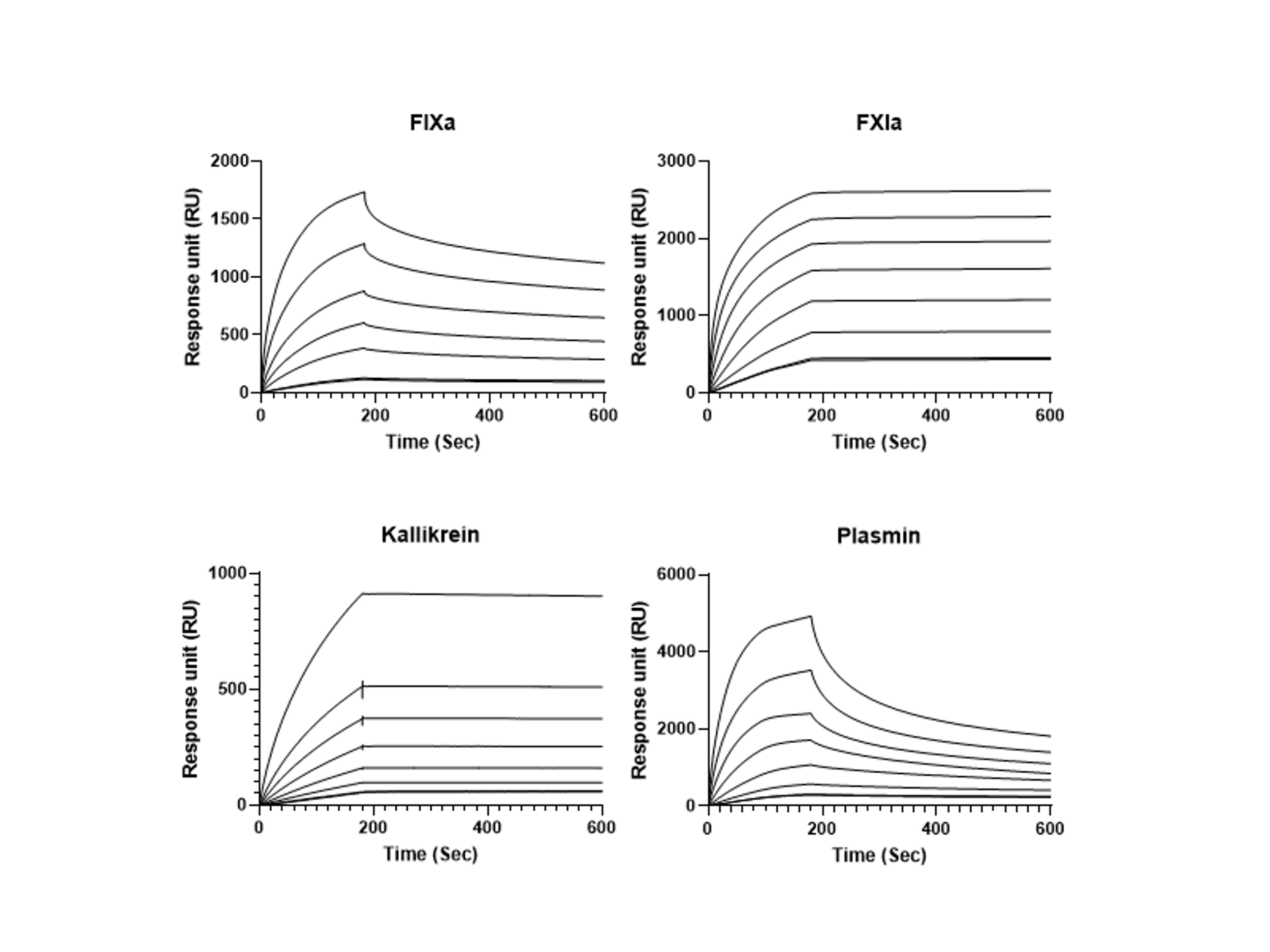


**Supplementary Figure 4: SPR-binding test of Guianensin and different coagulation factors.** Guianensin was immobilized on sensor CM5 chip, and factors FIXA, FXIa, Kallikrein, and Plasmin at 500 nM were flowed over the immobilized protein in 2-fold dilutions. For all SPR assays, the analytes were flowed over immobilized Guianensin at a contact time of 180 seconds, flow rate of 30µl/min, and dissociation time of 600 seconds. The sensor chip surface was regenerated by a pulse injection of 50 mm NaOH.

**Supplementary table 2:** Binding constant of Guianensin with XIa, Kallikrein, and Plasmin**.**

| **Analyte** | **K_a_ (m^-1^ s^-1^)** | **K_d_ (s^-1^)** | **K_D_ (M)** |
| --- | --- | --- | --- |
| IXa | 35415 ± 11476 | 0.001159 ± 0.0008679 | 3.872e-008 ± 3.703e-008 |
| XIa | 116733 ± 2831 | 4.505e-006 ± 4.508e-006 | 3.897e-011 ± 3.929e-011 |
| Kallikrein | 24800 ± 7562 | 2.339e-005 ± 6.690e-006 | 9.573e-010 ± 1.325e-010 |
| Plasmin | 136675 ± 109354 | 0.01021 ± 0.01220 | 5.742e-008 ± 4.354e-008 |

**
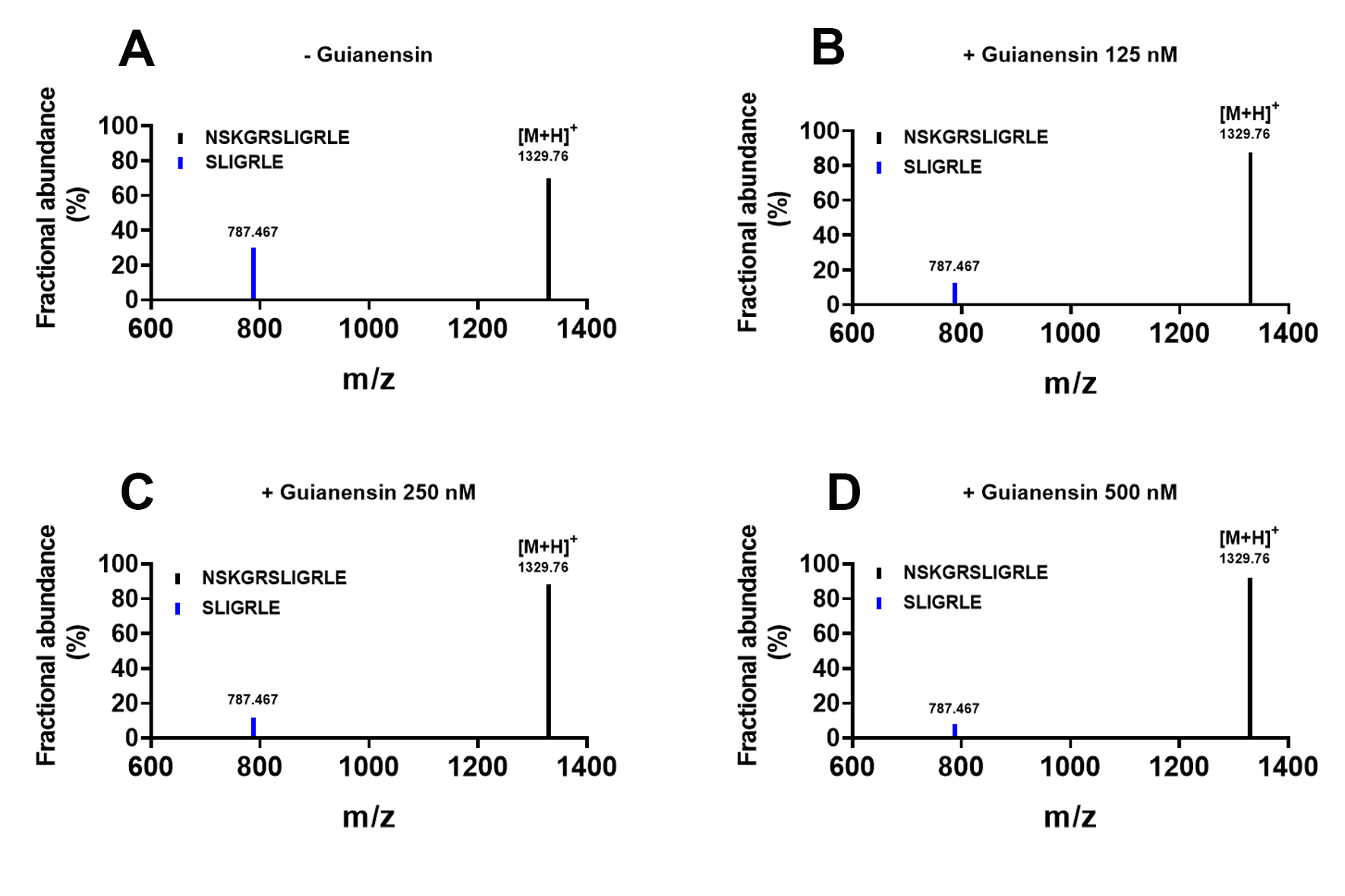
**

**Supplementary Figure 5: Guianensin inhibits FXa-induced PAR-2 activating peptide formation.** The PAR-2 cleavage products generated by FXa in the presence or absence of Guianensin was analyzed by ESI-MS. Deconvoluted mass spectra of full-length PAR-2 peptide (NSKGRSLIGRLE, MH+ monoisotopic mass 1329.7597 Da) and FXa cleavage product (SLIGRLE, MH+ monoisotopic mass 787.4672 Da) were detected. FXa (25 nM) was incubated at 37°C for 15 minutes with different concentrations of purified Guianensin (0, 125, 250, and 500 nM). PAR-2 (10 ug) was added in a final volume of 100 uL in PBS pH 7.4, and the reaction was incubated for 4 hours at 37°C. PAR-2 incubated with FXa **(A)** or FXa + Guianensin **(B-D)** were analyzed with a Q Exactive Plus Mass Spectrometer at 280k resolution. Mass spectra with less than 1% relative abundance, except for FXa cleavage product, are not shown in the figure.

**
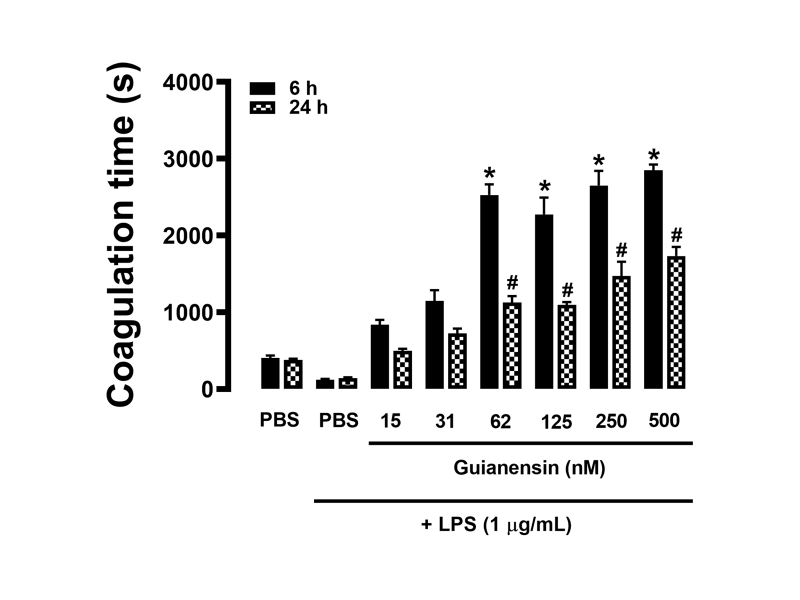
**

**Supplementary Figure 6:** **Guianensin inhibits LPS-induced procoagulant effect in human endothelial cells.** Confluent HMEC-1 were treated either 6 or 24 h with LPS to activate endothelial cells. Then, the coagulation time was measured after the addition of human plasma in the presence of Guianensin or PBS as a control. Data are presented as mean ± SE of coagulation time measured in seconds. The symbols (*) and (#) represent a statistically significant difference between Guianensin-treated groups versus PBS+LPS groups after 6 and 24 h of treatment, respectively (one-way ANOVA followed by Bonferroni’s post hoc test).
